# Assessment of Radiation Dosage to the Hippocampi during Treatment of Multiple Brain Metastases Using Gamma Knife Therapy

**DOI:** 10.3390/medicina60020246

**Published:** 2024-01-31

**Authors:** Maciej Laskowski, Bartłomiej Błaszczyk, Marcin Setlak, Maciej Kuca, Arkadiusz Lech, Kamil Kłos, Adam Rudnik

**Affiliations:** 1 Student Scientific Society, Department of Neurosurgery, Faculty of Medical Sciences in Katowice, Medical University of Silesia, 40-055 Katowice, Poland; s76438@365.sum.edu.pl (M.K.); s81021@365.sum.edu.pl (K.K.); 2 Department of Neurosurgery, University Clinical Center, Faculty of Medical Sciences in Katowice, Medical University of Silesia, 40-752 Katowice, Poland; bblaszczyk@sum.edu.pl (B.B.); marcin.setlak@sum.edu.pl (M.S.); adam.rudnik@sum.edu.pl (A.R.); 3 Exira Gamma Knife, 40-952 Katowice, Poland; a.lech@exira.pl

**Keywords:** gamma knife, radiation, hippocampus

## Abstract

*Background and Objectives:* Brain metastases (BMs) pose significant clinical challenges in systemic cancer patients. They often cause symptoms related to brain compression and are typically managed with multimodal therapies, such as surgery, chemotherapy, whole brain radiotherapy (WBRT), and stereotactic radiosurgery (SRS). With modern oncology treatments prolonging survival, concerns about the neurocognitive side effects of BM treatments are growing. WBRT, though widely used for multiple BMs, has recognized neurocognitive toxicity. SRS, particularly Gamma Knife (GK) therapy, offers a minimally invasive alternative with fewer side effects, suitable for patients with a quantifiable number of metastases and better prognoses. *Materials and Methods:* A retrospective analysis was conducted on 94 patients with multiple BMs treated exclusively with GK at an academic medical center. Patients with prior WBRT were excluded. This study focused on the mean radiation dose received by the hippocampal area, estimated according to the ‘Hippocampal Contouring: A Contouring Atlas for RTOG 0933’ guidelines. *Results:* The precision of GK equipment results in mean doses of radiation that are lower than those suggested by RTOG 0933 and observed in other studies. This precision may help mitigate cognitive dysfunction and other side effects of hippocampal irradiation. *Conclusions:* GK therapy facilitates the administration of smaller, safer radiation doses to the hippocampi, which is advantageous even for lesions in the temporal lobe. It is feasible to treat multiple metastases, including cases with more than 10, but it is typically reserved for patients with fewer metastases, with an average of 3 in this study. This underlines GK’s potential for reducing adverse effects while managing BMs effectively.

## 1. Introduction

The Central nervous system (CNS) is a frequent target for metastases from systemic cancer. About ten times as many people develop metastatic brain tumors as they do primary brain tumors [1]. The brain parenchyma and the leptomeningeal space are the most typical sites for CNS metastases [2], where 80% of metastatic lesions are supratentorial, and 20% are found in the posterior cranial fossa [3].

According to studies, 10% to 30% of cancer patients will acquire BMs (brain metastases) with lung, breast, colorectal, melanoma, and renal cell carcinoma being the most common causes [2,4]. The median survival rate for untreated brain metastases is as low as one month [5]. Some BMs are asymptomatic, whereas others exhibit symptoms similar to any intracranial space-occupying lesion linked to brain compression and mass effect. BMs patients can experience headaches, seizures, cognitive decline, fatigue, and focal deficits [6].

Up to 50% of all lung cancers eventually develop into brain metastases (BM), making the brain the most frequent site of lung cancer metastasis [7]. Lung cancers can be divided into small cell lung carcinoma (SCLC) and non-small cell lung carcinoma (NSCLC) based on the appearance and morphology of malignant cells [8]. A 50–80% likelihood of developing BMs is present in patients with small-cell lung cancer (SCLC) [9], which accounts for 15% of all lung malignancies [10]. Adenocarcinomas metastasis to the brain is more common than squamous cell carcinomas among non-small-cell lung malignancies (NSCLC). A total of 15% of all BMs are caused by breast cancer and the risk of metastasis is increased in estrogen receptor-negative and HER2/neu-positive tumors [11].

Multimodal therapies, which may combine surgery, radiation, chemotherapy, immunotherapy, and targeted medicines, are frequently used to treat BMs. The size, location, quantity, histopathology, and the extent of the primary tumor, as well as any prior anticancer therapies, all influence the choice of treatment method. Radiotherapy is the most effective nonsurgical treatment for treating brain tumors, and attempts are made to minimize any adverse effects that can have an impact on patients’ quality of life [12].

Concern over the side effects of treatment, particularly neurocognitive toxicity, has grown as patients are living longer after obtaining a diagnosis of and therapy for brain metastases. Depending on the radiation dose delivered to various brain areas during radiotherapy, other symptoms may also appear. According to the literature, there may be two primary pathogenic pathways for radiation-induced damage. The first one may be linked to damage to the endothelium of small blood arteries, which speeds up atherosclerosis and eventually results in chronic ischemia [13]. The direct injury of neuronal stem cells, specifically affecting those located in the hippocampus, may also be the cause of neurocognitive disorders caused by radiation [14]. DeAngelis’ study shows that within 5 to 36 months (median, 14) patients can develop progressive dementia, ataxia, and urinary incontinence, causing severe disability after WBRT therapy with the dosage of radiation estimated. In this study, the total dose of WBRT was only 2500 to 3900 cGy, but daily fractions were 300 to 600 cGy [15].

Over the past few decades, there have been considerable changes in how BMs are treated and managed, largely as a result of improvements in radiotherapeutic and neurosurgical methods. Resection, whole brain radiotherapy (WBRT), and stereotactic radiosurgery (SRS) have been the traditional therapeutic options, with very little historical use of chemotherapy. However, immune checkpoint inhibitors (ICI) and targeted agents with blood–brain barrier (BBB) penetration are expanding the use of systemic therapies in a subset of patients, and current research is concentrating on finding the most effective combinatorial strategies [16].

For patients with isolated, sizable, and surgically accessible brain metastases, neurosurgical excision is frequently the standard of care. The main benefit of resection is that it can effectively relieve symptoms and does so quickly. In patients with several cerebral lesions where one dominant lesion is generating a major mass effect that is life threatening or lowering quality of life, neurosurgical intervention is also a critical treatment. Regardless of the systemic therapy used, patients with symptomatic brain metastases should receive local therapy. Local therapy should not be postponed in patients with asymptomatic brain metastases unless this guideline specifically advises doing so. Delaying local therapy should be decided upon after a multidisciplinary discussion of the possible advantages and disadvantages for the patient [17].

Whole-brain radiation therapy (WBRT) has been the most widely used treatment for patients with multiple BMs, given its effectiveness in palliation and availability [18]. However, it is a palliative method used in symptomatic patients, aimed at improving their condition, and in whom it is impossible to use stereotaxic methods, e.g., due to too many neoplastic lesions. Radiation therapy carries the risk of nerve damage, including focal cerebral necrosis, brachial plexus neuropathy, cerebrovascular disease, and cognitive dysfunction [19]. Typical acute adverse effects of WBRT include temporary alopecia, mild dermatitis, mild fatigue, and less commonly, otitis media or externa. Significant verbal learning and memory impairment may result from high radiation exposure to the left hippocampus. Verbal fluency, executive function, and processing speed may all be impacted by high radiation doses to the left hippocampus and other left side structures, while processing speed and executive function may also be impacted by radiation to the thalamus [20]. The hippocampal region is thought to be responsible for preserving the neurocognitive functions in the human brain [21]. Cognitive degradation has been associated with radiation doses to the neurodegenerative zone of the hippocampus [22]. Even a dose of 2 Gy delivered to human neural stem cells could potentially decrease the number of cells that undergo neuronal differentiation [12,23]. According to studies, different subpopulations of stem and progenitor cells in the adult hippocampus respond differently to gamma radiation, and even after a temporary restoration, neurogenesis is impaired long term following gamma radiation exposure [24].

It is believed that cognitive function can be preserved by hippocampal avoidance (HA) during WBRT [21,22,25] by selectively restricting the radiation dose in the hippocampal region [26]. Patients who received HA-WBRT plus memantine compared favorably to those who received conventional WBRT plus memantine, with the former group reporting less memory loss, less difficulty speaking and using imputed data, less interference from neurologic symptoms in daily activities, and fewer cognitive symptoms. 22 Hippocampal avoidance and protective agents (such as memantine) could be used in daily clinical practice for the benefit of patients treated with prophylactic cranial irradiation (PCI), which has been regarded as the standard of care for the treatment of limited-stage and extensive-stage small-cell lung cancer (LSCLC), to help alleviate the symptoms of potential hippocampal deterioration [27]. Le Fèvre’s (2021) study suggests that radiation dosage and a decline in hippocampus volume are correlated. The hippocampi, however, appear to show an adaptive increase in their volume at the lowest doses, which may point to a plasticity effect. Therefore, it is advised to shield at least one hippocampus by administering the lowest dose possible in order to maintain cognitive function [28]. In RTOG 0933, a phase II clinical trial, the possibility of reducing radiation-induced neurocognitive damage by avoiding cranial irradiation of the hippocampus was investigated [29]. The RTOG 0933 hippocampal dose criteria for hippocampal avoidance (dose to 100% of the hippocampus could not exceed 9 Gy, and maximal hippocampal dose could not exceed 16 Gy) [25] could be achieved using current IMRT methods, which spare the hippocampus while providing appropriate target coverage and homogeneity [29]. The dose criteria from the RTOG 0933 study and further NRG Oncology CC001 phase III trial are often used as a reference in modern research concerning hippocampus sparing but, before incorporating the hippocampus sparing concept into standard therapeutic practice, more research is likely to be required [30]. 30 The recent Goda study from 2020 concerning long-term cognitive outcomes in young patients with brain tumors suggests that hippocampal doses (>30 Gy mean dose to the left hippocampus) seem to be strongly correlated with IQ loss and recommends a mean dose of ≤30 Gy to the left hippocampus as a dose constraint for preserving intelligence quotient, because a higher dose to the hippocampi may result in long-term neurocognitive impairment. This dose could be relatively simpler to achieve in standard clinical practice than the RTOG 0933 criteria. Considering that the hippocampal dose in this study is much higher than the RTOG 0933 suggests, further evaluation in the future may be required. Due to study findings, even low-dose stereotactic radiation using LINAC technology delivered directly to the temporal lobe may cause long-term neurological issues such as significant IQ decline, memory loss, partial aphasia, hemiplegia, progressive ataxia, and cognition decline [31].

Stereotactic radiosurgery may be an appropriate alternative for patients with up to ten brain metastases due to its minimal invasiveness and lack of side effects compared to WBRT [32]. Leksell’s concept and development of stereotactic radiosurgery (SRS) is based on delivering 192 convergent beams of gamma radiation emitted from cobalt-60, directed toward a central point to offer conformal delivery of a high dose of radiation to a specific tumor volume within the brain and to prevent administering high-dose irradiation to nerve tissue [33]. To model radiation isodose lines and define a radiation dose to the tumor target, three-dimensional treatment plans can be created using the GK software. The radiation dosage rapidly decreases outside the tumor target’s volume, preventing the delivery of significant radiation doses to the healthy brain [34], which offers the chance to provide patients with individualized care. GK permits retaking sessions while minimizing procedural uncertainty, taking into account numerous challenges during therapy and the necessity of reassessing the exposure to massive, lethal doses of radiation administered frequently in close proximity to healthy, radiosensitive nervous system tissue [35]. GK can be considered as an effective treatment for patients with numerous brain metastases based on the previously disclosed characteristics and clinical experiences with multiple metastases within the CNS.32 Compared to WBRT, Gamma Knife exhibits significantly better results and less toxicity [36]. According to studies, individuals with brain metastases who have Gamma Knife SRS have an average local control rate of 84–97%. It is also important to note that surgically removed metastases require postsurgical adjuvant SRS because surgical resection alone has a 46% local recurrence risk [37]. According to prior studies examining the use of GK in the treatment of metastases found in the temporal lobe, GK therapy may help prevent unintended neurological dysfunction caused by nonspecific radiation delivery to eloquent nerve tissues while maintaining the efficacy of WBRT [38]. In a carefully selected group of oncological patients, GK was also used in the reirradiation of metastatic foci. However, in such a clinical scenario, it is worth taking into account that repeat radiosurgery of recurrent lesions greatly exacerbates the risk of radiation necrosis [39]. It is worth mentioning that GK was used in patients in good general condition for whom we plan to follow longer. WBRT is a palliative treatment used to reduce symptoms caused by the compression of the total mass of tumors. WBRT and GK are not competing methods, as they were used in different groups of patients—GK in the case of patients with a quantifiable number of metastases, in good condition, with whom we expected a longer survival period, and WBRT as a palliative method for symptomatic patients with a worse prognosis or, with a very high or even uncountable number of metastases [40].

## 2. Materials and Methods

### 2.1. Patient Selection, Inclusion and Exclusion Criteria

**Figure 1:**
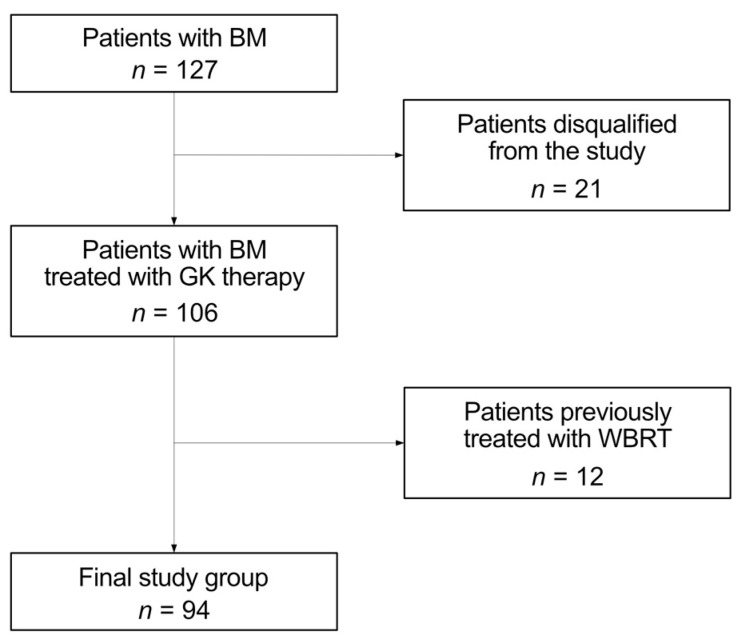
Final group creation process.

We performed a retrospective analysis of all patients diagnosed with multiple BMs admitted to the Exira Gamma Knife of the Voxel S.A. in Katowice, Poland, from January 2021 to June 2022. Clinical information about the patients was registered and documented, including age, sex, primary oncological disease, number of GK therapies, number of metastases, information on whether the metastasis was located in the temporal lobe, information on whether the patient underwent WBRT therapy before GK therapy, and information on whether patient received chemotherapy to treat a primary tumor. The initial group consisted 127 individuals. We excluded all patients without complete data or follow-up information (n = 21). The analysis concerned patients treated only with GK therapy, so patients previously treated with WBRT also were excluded (n = 12). Our objective was to exclusively assess the dosage administered during GK therapy, as prior WBRT could potentially alter the outcomes. Table 1 shows histopathological diagnosis, number, percentage, mean ages, and sex distribution of our patients with BMs. The final study group consisted of 94 patients, 59 (62.8%) females and 35 (37.2%) males, with an average age of 64 (range 37–82). The process of the final group creation is presented in Figure 1.

### 2.2. Indications for the Gamma Knife Therapy

Patients chosen for Gamma Knife therapy had numerous, numerable brain metastases, up to 10, each with a diameter under 3 cm. These individuals were in good general health, maintaining a Karnofsky performance status (KPS) of 70 or above. The study focused on patients with multiple brain metastases situated beyond a 5-mm margin from the hippocampus and with a histopathologicaly confirmed diagnosis of nonhematopoietic malignancy.

### 2.3. Gamma Knife Therapy

All patients underwent GK treatment using a Leksell Gamma Knife^®^ Perfexion^TM^ (Elekta Instrument AB, Stockholm, Sweden). Prior to the treatment patients underwent comprehensive preparation, which included a detailed neurological assessment to evaluate their suitability for the procedure. A stereotactic frame was attached to the patient’s skull after local anesthesia. Treatment planning was performed using Leksell GammaPlan ver. 11.1.1 software (Elekta AB). BMs were defined on 1-mm thickness gadolinium-enhanced T1-weighted images, T1-weighted images with Contrast, T2-weighted, and Fluid attenuated inversion recovery (FLAIR) along with Magnetization transfer (MT) imaging. After positioning, confirmed by high-resolution imaging, GK radiosurgery was performed.

### 2.4. Post-Treatment Management

Two months post Gamma Knife surgery, patients underwent a follow-up T1-weighted MRI, a T1-weighted MRI with Contrast, a T2-weighted MR, and FLAIR along with MT imaging to assess tumor regression and clinical status. Subsequent MRI monitoring was scheduled every 2–3 months using MRIs (T1-weighted, T1-weighted with Contrast, T2-weighted, FLAIR along with MT), alongside evaluations at the originating oncology centers. For tumors larger than 10 cm^3^, a dosage of 14 Gy was administered with a follow up after three weeks. If there was a positive response to treatment, as evidenced by a reduction in tumor mass, patients were considered for additional radiosurgical intervention.

### 2.5. Assessing Radiation Dose to Hippocampi and Contouring Process

In each patient we estimated the mean dose of radiation received in this therapy of the hippocampal area on both sides by outlining the hippocampal area in many layers of MR scans. The outlining was performed with Leksell GammaPlan ver. 11.1.1. We followed Hippocampal Contouring: A Contouring Atlas for RTOG 0933 guidelines.

Contouring the hippocampus according to the RTOG 0933 guidelines involves a meticulous step-by-step process to ensure precision. The process begins by anatomically defining the hippocampal region, identifying landmarks such as the lateral ventricle, and encompassing the entire hippocampal formation, including the head, body, and tail.

For optimal visualization, high-resolution T1-weighted MRI is recommended. The contouring occurs slice-by-slice on axial images, extending the delineation along the entire length of the hippocampus in each slice. Throughout this process, it is crucial to exclude adjacent structures while capturing the complete hippocampal formation.

Special attention is given to avoiding structures like the choroid plexus and vascular elements within the hippocampal formation during contouring. Quality assurance measures are implemented to validate the contours, ensuring accuracy and reproducibility. This standardized approach enhances consistency across different practitioners and institutions, contributing to the reliability of research and clinical trials involving treatments like Gamma Knife therapy for brain metastases.

**Figure 2:**
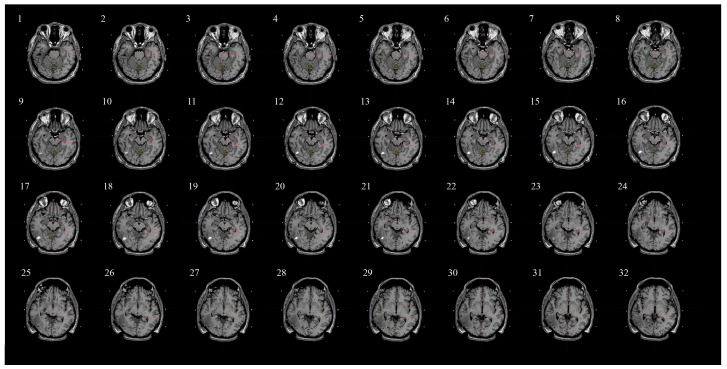
An example illustrating the sections where hippocampal outlines were contoured. The outlines of the hippocampi are marked with red lines. Cross-sectional slices in the T1-weighted MRI sequence, differing from each other by 1.25 mm.

Figure 2 illustrates the sections on which we outlined the hippocampi (contours marked in red) in one of the patients. As the Contouring Atlas suggests, we used 1.25 mm slice thickness layers to contour the hippocampus accurately. Contouring was performed only on T1-weighted MRI axial sequences. This enabled the Leksell GammaPlan ver. 11.1.1 software (Elekta, Stockholm, Sweden) creation of a model of the hippocampus and, based on its volume, calculation of the mean radiation dosage in this specific area.

Statistical analysis of the collected data was conducted using Statistica 13.0 software (StatSoft, Krakow, Poland). Categorical variables are described by numbers and per-centages while quantitative variables are described by the mean and its confidence in-terval or median and interquartile range. Normality of the distribution of obtained results was assessed using a Shapiro–Wilk test.

## 3. Results

Presenting the outcomes of our analysis in Table 2 and Table 3, we conducted a study on patients admitted to the Exira Gamma Knife of Voxel S.A. in Katowice, Poland, for the treatment of brain metastases. Table 1 provides insights into the number of therapies and brain metastases in the study group. The median number of Gamma Knife therapies was 2, ranging from a minimum of 1 to a maximum of 9, while the median number of brain metastases was 3, with a minimum of 2 and a maximum of 20. In Table 3, we detail the radiation doses to the left hippocampus, right hippocampus, and bilateral hippocampi. The maximum evaluated radiation dosage was 7 Gy (±1.1) in the right hippocampus, 6.9 Gy (±1.2) in the left hippocampus, and 8.3 Gy (±1.8) in the bilateral hippocampi. Conversely, the mean dosage was 0.9 Gy (±1.2) in the left hippocampus, 0.81 Gy (±1.1) in the right hippocampus, and 1.66 Gy (±1.8) in the bilateral hippocampi. These findings contribute to a comprehensive understanding of our research results intended for inclusion in the medical article.

In Table 4, the same results were presented for patients who had metastases in the temporal lobe, i.e., in close proximity to the hippocampi. As indicated by the calculations, the radiation doses within the hippocampal region were slightly higher.

## 4. Discussion

Radiosurgery with the use of GK allows for a significant dose reduction in the hippocampal area, even in patients with multiple metastases to the brain (even more than 10 metastases). Thanks to the extremely precise technique of planning and implementing radiotherapy, we obtained a high dose gradient outside the irradiated area, which allowed for the protection of eloquent structures of the brain while depositing a therapeutic dose in the range of 18 to 24 Gy to the tumor. Qualification for reradiosurgery in the cerebral area should be undertaken consciously based on interdisciplinary teams in centers with extensive experience in the field of stereotactic radiotherapy.

GK is an effective alternative to conformal radiotherapy of the whole brain, with the preservation of hippocampal structures in patients with good performance status and quantifiable metastatic changes, allowing for reduced risk of radiation-induced cognitive disorders and better local control (higher ranges of biologically effective doses). Patients after radiosurgery require close oncological supervision with periodic (2–3 months) MR imaging in order to monitor potential subclinical metastatic foci and initiate adequate treatment early. Table 5 showed cases from our study within and exceeding suggested hippocampal avoidance doses from the literature. Mean doses of radiation from our research compared to the suggested doses from RTOG 0933 and other studies are relatively low due to the precision that can be achieved with GK equipment, which could possibly reduce damage to cognitive functions and other side effects associated with irradiation of the hippocampi. In our research we decided to evaluate only the mean doses of radiation. Even though RTOG 0933 recommends a maximal hippocampal dose of ≤16 Gy, the mean dose of radiation delivered to the structure of the hippocampus better represents the possible damage that can be done during the GK procedure due to the fact that the hippocampus is a relatively large structure. This fact is also represented by the latest study by Goda from 2020, that suggests only a mean dose of ≤30 Gy to the left hippocampus for preserving cognitive functions and Gondi’s previous research from 2011 which recommends EQD(2) to 40% of the bilateral hippocampi of less than 7.3 Gy for hippocampal avoidance. It is worth mentioning that GK was used in patients in good general condition of whom we expected to live longer, therefore it was of great importance to preserve patients’ cognitive functions and improve their quality of life instead of worsening it. Even though over half of the patients in this study (64.9%) had a lesion located in the temporal lobe, the dosage of radiation delivered to both hippocampi remained within the hippocampal avoidance recommendations from the literature for almost all treated cases included in this study.

During our research we also made a detailed comparison of the results from our findings and relevant studies from the past regarding hippocampal avoidance. According to the meta-analysis concerning hippocampal sparing radiation therapy for brain metastases performed by Goods in 2023, in all analyzed studies which used GK technology in treatment of the brain metastases the mean dose to hippocampus was within RTOG 0933 criteria and the mean doses were almost identical to our findings [42]. It is also worth mentioning that GK methods can considerably reduce doses to the hippocampi compared with HA-WBRT by more than 80%, even without specifically avoiding the hippocampi [43]. Additionally, in the majority of situations, hippocampal sparing can be easily accomplished with GK even without taking hippocampal avoidance into deliberate consideration [44]. Taking into consideration these findings in terms of hippocampal avoidance, GK can be an excellent tool for preserving patients’ cognitive functions. However, the direct comparison of the analyzed studies proved a significant challenge due to very significant disparities in methodology, doses used in treatments, numbers of metastases, contouring of hippocampus, and GK techniques used by different authors; therefore, for comparison purposes, we decided to rely only on the mean dose to hippocampus which was the most objective value.

WBRT is a palliative treatment used to reduce symptoms caused by the compression of the total mass of tumors. It is reserved for patients with symptomatic and multiple dissemination of neoplastic disease to the brain with a significant reduction in overall efficiency. In a strictly defined clinical situation, both methods of radiotherapy (WBRT and RS) can be used sequentially. Both methods are reserved for separate patient groups. We used GK in patients with a limited number of meta, in good performance status, and with a limited volume of target areas. In this technique, we administered higher biological doses. One fraction of GK lasts a much shorter time than WBRT, which is important in the case of pain patients who have problems with maintaining the supine position.

HA-WBRT is a recommended elective treatment option for patients with good prognosis and multiple brain metastases as it results in better neurocognitive preservation compared to whole brain radiotherapy. HA-WBRT performed with the use of modern techniques of dynamic radiotherapy (IMRT, VMAT) also allows for a significant dose reduction in the hippocampus area; however, due to the scope of the irradiated area, the average doses will be higher than in stereotaxic techniques. Unfortunately, this method is not commonly used in daily clinical practice.

## 5. Conclusions

GK allows us to deliver smaller, safer doses to the hippocampi, even if the lesion is located in the temporal lobe. It is also possible to treat patients with multiple metastases (even more than 10 metastases at the same time), but more often this method is used in patients with fewer metastases—in our case an average of 3. The GK method enables multiple treatments, even in a short period of time, with a high chance of preserving patients’ cognitive function after treatment, unlike WBRT, which is a one-time palliative treatment. WBRT has an advantage for patients with a large number of minor metastases, while with GK, very small changes may not be detected on an MRI.

## Figures and Tables

**Table 1:** Histopathological diagnosis, number, percentage, mean ages, and sex distribution of our patients with BMs.

Histopathological Diagnosis	Frequency, n (%)	Mean Age (Years)	Male/Female
Non-Small Cell Lung Cancer	53 (56.4)	66.3	25|28
Small Cell Lung Cancer	5 (5.3)	61.8	1|4
Breast Cancer	21 (22.3)	62.7	0|21
Clear-cell Renal Cell Carcinoma	4 (4.3)	70	4|0
Adenocarcinoma Colon Cancer	4 (4.3)	58.5	2|2
Melanoma	2 (2.1)	54	1|1
Ovarian Cancer	2 (2.1)	73.5	0|2
Urothelial Carcinoma	1 (1.1)	70	1|0
Gastric Cancer	1 (1.1)	50	1|0
Cervical Cancer	1 (1.1)	67	0|1
All	94 (100)	63.6	35|59

**Table 2:** Number of therapies and number of metastases of patients in the study group.

	Number of Therapies	Number of Metastases
N-valid	94	94
N-missing	0	0
Median	2	3
Minimum	1	2
Maximum	9	20

**Table 3:** Gamma Knife mean radiation doses to the left hippocampus, right hippocampus, and bilateral hippocampi in all patients of our study.

	N	Minimum	Maximum	Mean	Std. Deviation
Radiation dose to the left hippocampus	94	0.0	6.9	0.906	1.2517
Radiation dose to the right hippocampus	94	0.0	7.0	0.806	1.0779
Radiation dose to the bilateral hippocampi	94	0.0	8.3	1.713	1.7964

**Table 4:** Gamma Knife mean radiation doses to the left hippocampus, right hippocampus, and bilateral hippocampi in patients that had temporal lobe metastases.

	N	Minimum	Maximum	Mean	Std. Deviation
Radiation dose to the left hippocampus	66	0.0	6.9	1.068	1.4096
Radiation dose to the right hippocampus	66	0.0	7.0	0.968	1.2188
Radiation dose to the bilateral hippocampi	66	0.1	8.3	2.036	0.2394

**Table 5:** Number of cases from our study within and exceeding suggested hippocampal avoidance doses from the literature. EQD(2)—equivalent dose in 2 Gy fractions.

Name of the Study	Hippocampal Avoidance Recommendation	Number of Cases within the Limit	Number of Cases Exceeding the Limit
Goda (2020) [21]	Mean dose of ≤30 Gy to the left hippocampus	94	0
NRG Oncology CC001 (2020) [22]	Dose to 100% of the hippocampus ≤9 Gy, maximal hippocampal dose ≤16 Gy	94	0
RTOG 0933 (2014) [25]	Dose to 100% of the hippocampus ≤9 Gy, maximal hippocampal dose ≤16 Gy	94	0
Gondi (2012) [41]	EQD(2) to 40% of the bilateral hippocampi lesser than 7.3 Gy	92	2

## Data Availability

No new data were created or analyzed in this study. Data sharing is not applicable to this article.
